# Supplementary material for: Content-rich biological network constructed by mining PubMed abstracts
Source: BMC Bioinformatics. 2004 Oct 8;5:147. doi: 10.1186/1471-2105-5-147 (PMC528731; doi:10.1186/1471-2105-5-147)
Supplement: Additional File 2 — The original results of the above study (non-essential files are deleted to keep the file size under the limit set by BMC bioinformatics). [file 1471-2105-5-147-S2.bz2 › chilibotAdditionalFile2/dip40/674ID10767348E11080/html/ACVRL1_ENG.html]

 


 **ACVRL1** and **ENG** 
  
Found 524 abstracts in PubMed, retrieved 40.  
 

 What does Google say? 
 PDF only 
| .edu only 

---

**Interactive relationship** (e.g. stimulation, inhibition, etc)

**Neutral relationship**- Thromboxane A 2 synthase TXAS binds to the endoplasmic reticulum membrane and catalyzes both an isomerization of prostaglandin H 2 PGH 2 to form thromboxane A 2 TXA 2 and a fragmentation of PGH 2 to form 12 hydroxy, 10 heptadecatrienoic acid HHT  [ **ENG** ]   [ **ACVRL1** ]  and malondialdehyde MDA.  Ref: 12859980 Arch Biochem Biophys, 2003
- We found that HHT  [ **ENG** ]   [ **ACVRL1** ]  was metabolized to an acid product HHT  [ **ENG** ]   [ **ACVRL1** ]  acid.  Ref: 12724858 J Exp Ther Oncol< MedlineTA>J Exp Ther Oncol, 2003
- HHT  [ **ENG** ]   [ **ACVRL1** ]  types 1 and 2 are caused by loss of function mutations in  **ENG**  and  **ACVRL1** .  Ref: 12812985 Hum Mol Genet, 2003
- Activin receptor like kinase 1  [ **ACVRL1** ]  ALK 1  [ **ACVRL1** ] , the gene mutated in HHT  [ **ENG** ]   [ **ACVRL1** ]  type 2 HHT2  [ **ACVRL1** ] , is a serine threonine kinase receptor type I of the TGF beta superfamily, specifically expressed on endothelial cells.  Ref: 12700602 Eur J Hum Genet, 2003
- The two known disease types, HHT1  [ **ENG** ]  and HHT2  [ **ACVRL1** ] , are caused by mutations in the endoglin  [ **ENG** ]   **ENG**  and ALK 1  [ **ACVRL1** ]  genes, respectively.  Ref: 12843319 J Med Genet< MedlineTA>J Med Genet, 2003
- Should asymptomatic patients with hereditary haemorrhagic telangiectasia HHT  [ **ENG** ]   [ **ACVRL1** ]  be screened for cerebral vascular malformations ? Data from 22 061 years of HHT  [ **ENG** ]   [ **ACVRL1** ]  patient life.  Ref: 12754343 J Neurol Neurosurg Psychiatry, 2003
- HHT  [ **ENG** ]   [ **ACVRL1** ]  type 2 is caused by loss of function mutations in activin receptor like kinase 1  [ **ACVRL1** ]   **ACVRL1**  or ALK1  [ **ACVRL1** ] .  Ref: 12588795 Hum Mol Genet, 2003
- Moreover, mutations in the genes encoding H3 HHT1  [ **ENG** ]  and HHT2  [ **ACVRL1** ]  and H4 HHF1 and HHF2 mapping to spatially adjacent amino acid residues affected the three forms of silencing distinctly, suggesting that specific interactions mediate each form of silencing.  Ref: 12244315 Nat Genet, 2002
- In mice, HHT  [ **ENG** ]   [ **ACVRL1** ]  acid was detected in the plasma within 5 min of the i.v. injection of HHT  [ **ENG** ]   [ **ACVRL1** ]  and declined rapidly thereafter.  Ref: 12724858 J Exp Ther Oncol< MedlineTA>J Exp Ther Oncol, 2002
- Hereditary hemorrhagic telangiectasia HHT  [ **ENG** ]   [ **ACVRL1** ]  is a vascular disorder in humans which has been mapped to two genes, endoglin  [ **ENG** ]  and activin receptor like kinase 1  [ **ACVRL1** ]  ALK 1  [ **ACVRL1** ]  both of which mediate signaling by transforming growth factor beta ligands in vascular endothelial cells.  Ref: 12667943 Cardiovasc Res, 2003
- Twenty four hours after HHT  [ **ENG** ]   [ **ACVRL1** ]  dosing in mice, approximately 29% of the dose was excreted in the urine as HHT  [ **ENG** ]   [ **ACVRL1** ]  and 20% as HHT  [ **ENG** ]   [ **ACVRL1** ]  acid.  Ref: 12724858 J Exp Ther Oncol< MedlineTA>J Exp Ther Oncol, 2003
- The similarity of affected organs, age dependent penetrance, histological similarity of the lesions and recapitulation of a secondary phenotype suggest that the  **Acvrl1**  mice are an appropriate animal model for the identification of additional genetic and environmental factors that cause pathology in HHT  [ **ENG** ]   [ **ACVRL1** ]  type 2 patients.  Ref: 12588795 Hum Mol Genet, 2003
- In contrast, ALK 1  [ **ACVRL1** ]  functions as a TGF beta type I receptor in endothelial cells, and is responsible for human hereditary hemorrhagic telangiectasia HHT  [ **ENG** ]   [ **ACVRL1** ]  type II.  Ref: 12384983 J Cell Physiol, 2002

**Non-interactive relationship** (e.g. studied together, co-existance, homology, etc.)

- The HHT  [ **ENG** ]   [ **ACVRL1** ]  concentration inhibiting 50% of the growth of human leukemic HL 60 cells was 20 ng ml, while for HHT  [ **ENG** ]   [ **ACVRL1** ]  acid it was 1 00 ng ml, indicating that the acid form was more than 700 times less cytotoxic than HHT  [ **ENG** ]   [ **ACVRL1** ] .  Ref: 12724858 J Exp Ther Oncol< MedlineTA>J Exp Ther Oncol, 2002
- OBJECTIVE Our aim was to report our experience with treating GI bleeding in patients with hereditary hemorrhagic telangiectasia HHT  [ **ENG** ]   [ **ACVRL1** ] .  Ref: 12526937 Am J Gastroenterol, 2003
- 1.05 to 2.99 yielded a haemorrhage rate in individuals with cerebral AV malformations of 1.4 2.0% per annum, comparable to figures in the non HHT  [ **ENG** ]   [ **ACVRL1** ]  cerebral AV malformation population.  Ref: 12754343 J Neurol Neurosurg Psychiatry, 2003
- HHT  [ **ENG** ]   [ **ACVRL1** ]  is inherited as an autosomal dominant trait and there is no age cut off when apparently unaffected offspring of an individual with HHT  [ **ENG** ]   [ **ACVRL1** ]  can be told they are unaffected.  Ref: 12566546 Postgrad Med J, 2003
- Methods We reviewed the angiographic findings of seven patients with HHT  [ **ENG** ]   [ **ACVRL1** ] .  Ref: 12813620 Cardiovasc Intervent Radiol< MedlineTA>Cardiovasc Intervent Radiol, 2003
- Inhibition concentration 50% values of CET and HHT  [ **ENG** ]   [ **ACVRL1** ]  were significantly correlated to doxorubicin, vincristine, methotrexate, cisplatin, or camptothecin in 55 cell lines of the Developmental Therapeutics Program of the National Cancer Institute.  Ref: 12616342 Naunyn Schmiedebergs Arch Pharmacol, 2003
- PATIENTS AND METHODS 69 patients with HHT  [ **ENG** ]   [ **ACVRL1** ]  were treated with APC and estriol nose ointment.  Ref: 12589417 HNO, 2003
- RESULTS The study included 43 HHT  [ **ENG** ]   [ **ACVRL1** ]  patients with a mean age of 57 yr.  Ref: 12526937 Am J Gastroenterol, 2003
- The point prevalence of HHT  [ **ENG** ]   [ **ACVRL1** ]  in the Afro Caribbean population of the Netherlands Antilles is the highest known in the world.  Ref: 12522784 Am J Med Genet, 2003
- Hereditary hemorrhagic telangiectasia HHT  [ **ENG** ]   [ **ACVRL1** ]  is associated with arteriovenous malformation in multiple organs.  Ref: 12517049 J Formos Med Assoc, 2002
- The association of HHT  [ **ENG** ]   [ **ACVRL1** ]  with primary malignancy has rarely been reported.  Ref: 12517049 J Formos Med Assoc, 2002
- AHT 1 and Rhizomucor pusillus HHT  [ **ENG** ]   [ **ACVRL1** ]  1.  Ref: 12201132 Water Sci Technol, 2002
- The identification of ALK 1  [ **ACVRL1** ]  mutations in subjects with a suspected diagnosis and without clinical signs of HHT  [ **ENG** ]   [ **ACVRL1** ]  argue in favour of a molecular diagnosis.  Ref: 12843319 J Med Genet< MedlineTA>J Med Genet, 2002
- Purpose To describe the angiographic features of hepatic involvement in hereditary hemorrhagic telangiectasia HHT  [ **ENG** ]   [ **ACVRL1** ] , particularly the presence of portovenous shunts.  Ref: 12813620 Cardiovasc Intervent Radiol< MedlineTA>Cardiovasc Intervent Radiol, 2002
- STUDY OBJECTIVES Pulmonary arteriovenous malformations PAVMs in patients with hereditary hemorrhagic telangiectasia HHT  [ **ENG** ]   [ **ACVRL1** ]  can cause hemorrhage, stroke, and cerebral abscess.  Ref: 12576351 Chest, 2003
- In conclusion, the transcription of hTERT mRNA in HL 60 cells can be inhibited by HHT  [ **ENG** ]   [ **ACVRL1** ] .  Ref: 12744738 Zhongguo Shi Yan Xue Ye Xue Za Zhi, 2003
- BACKGROUND AND OBJECTIVE Hereditary hemorrhagic telangiectasia HHT  [ **ENG** ]   [ **ACVRL1** ]  is an autosomal dominant fibrovascular dysplasia with the main symptom of recurrent epistaxis.  Ref: 12589417 HNO, 2003
- We correlated the IC values of CET and HHT  [ **ENG** ]   [ **ACVRL1** ]  with the cell doubling times of the 55 NCI cell lines as proliferation parameter and observed that rapidly growing cells were more susceptible than slowly growing cell lines.  Ref: 12616342 Naunyn Schmiedebergs Arch Pharmacol, 2003
- Hepatic involvement in hereditary hemorrhagic telangiectasia HHT  [ **ENG** ]   [ **ACVRL1** ]  is highly variable and may lead to severe clinical symptoms such as heart failure.  Ref: 12717395 Hepatology, 2003
- The Harmonic Scalpel may be the treatment of choice for recurrent epistaxis in HHT  [ **ENG** ]   [ **ACVRL1** ] .  Ref: 12658738 Head Neck, 2003
- Hereditary hemorrhagic telangiectasia HHT  [ **ENG** ]   [ **ACVRL1** ]  has been reported rarely in people of African descent.  Ref: 12522784 Am J Med Genet, 2003
- We conducted a search mining the NCI s database for the mRNA expression of 465 genes in 55 cell lines and correlated the data with the IC values for CET and HHT  [ **ENG** ]   [ **ACVRL1** ] .  Ref: 12616342 Naunyn Schmiedebergs Arch Pharmacol, 2003
- HHT  [ **ENG** ]   [ **ACVRL1** ]  inhibited tumor cell growth at molar ranges comparable to established cytostatic drugs.  Ref: 12616342 Naunyn Schmiedebergs Arch Pharmacol, 2003
- Of these genes 61 correlated with the IC values for CET and 122 with the IC values for HHT  [ **ENG** ]   [ **ACVRL1** ]  indicating the multifactorial mode of action of these drugs in cancer cells.  Ref: 12616342 Naunyn Schmiedebergs Arch Pharmacol, 2003
- We conclude that Ara C, DNR or HHT  [ **ENG** ]   [ **ACVRL1** ] , either alone or in combination with imatinib, are likely to be the best therapeutic alternatives in the management of patients who become resistant to imatinib monotherapy.  Ref: 12454739 Leukemia, 2002
- Conclusions Hepatic vascular lesions in HHT  [ **ENG** ]   [ **ACVRL1** ]  are varied, ranging from telangiectasias to large shunts between three vascular channels.  Ref: 12616420 Cardiovasc Intervent Radiol< MedlineTA>Cardiovasc Intervent Radiol, 2002
- We report two cases of HHT  [ **ENG** ]   [ **ACVRL1** ]  in which recurrent nasal bleeding was successfully controlled using the Harmonic Scalpel.  Ref: 12658738 Head Neck, 2003
- Homoharringtonine HHT  [ **ENG** ]   [ **ACVRL1** ] , first isolated from the Chinese evergreen Cephalotaxus harringtonia, has been demonstrated to have a broad antitumor activity in rodents and antileukemic effects in humans.  Ref: 12724858 J Exp Ther Oncol< MedlineTA>J Exp Ther Oncol, 2003
- Substrate specificity of the alpha L arabinofuranosidase from Rhizomucor pusillus HHT  [ **ENG** ]   [ **ACVRL1** ]  1.  Ref: 12829392 Carbohydr Res, 2003
- We found that these drugs are generally capable of major inhibition of proliferation of the CML cell lines, although differential responses to DNR and HHT  [ **ENG** ]   [ **ACVRL1** ]  were noted between some sensitive and resistant cell line pairs, implying that resistance to imatinib may confer a growth advantage under such conditions.  Ref: 12454739 Leukemia, 2002
- A higher frequency of pulmonary arteriovenous malformations AVMs has been reported for HHT1  [ **ENG** ]  while HHT2  [ **ACVRL1** ]  is thought to be associated with a lower penetrance and milder disease manifestations.  Ref: 12843319 J Med Genet< MedlineTA>J Med Genet, 2002
- In hereditary hemorrhagic telangiectasia HHT  [ **ENG** ]   [ **ACVRL1** ] , the hallmark vascular lesion is termed an arteriovenous malformation, which involves the direct communication of an artery with a vein arteriovenous shunt, without an intervening capillary bed.  Ref: 12668602 Hum Mol Genet, 2003
- Hereditary haemorrhagic telangiectasia HHT  [ **ENG** ]   [ **ACVRL1** ]  affects one in 5 8000, and no longer can be viewed as solely causing anaemia due to nasal and gastrointestinal bleeding and characteristic mucocutaneous telangiectasia.  Ref: 12566546 Postgrad Med J, 2003
- These findings have important implications for the follow up and management of HHT  [ **ENG** ]   [ **ACVRL1** ]  patients.  Ref: 12576351 Chest, 2003
- The results showed that HHT  [ **ENG** ]   [ **ACVRL1** ]  induced K562 cells to apoptotic death at the concentrations of 5 20 ng ml, and some of the cells became necrotic when exposed to a higher concentration.  Ref: 12578668 Zhongguo Shi Yan Xue Ye Xue Za Zhi< MedlineTA>Zhongguo Shi Yan Xue Ye Xue Za Zhi, 2003
- Purpose To describe the angiographic features of hepatic involvement in hereditary hemorrhagic telangiectasia HHT  [ **ENG** ]   [ **ACVRL1** ] , particularly the presence of portovenous shunts.  Ref: 12616420 Cardiovasc Intervent Radiol< MedlineTA>Cardiovasc Intervent Radiol, 2003
- In this study, the underlying antileukemic mechanisms of homoharringtonine HHT  [ **ENG** ]   [ **ACVRL1** ]  were investigated.  Ref: 12578668 Zhongguo Shi Yan Xue Ye Xue Za Zhi< MedlineTA>Zhongguo Shi Yan Xue Ye Xue Za Zhi, 2003
- Four paired imatinib sensitive resistant CML cell lines were assessed by caspase 3 and MTS assays for their proliferative response to cytosine arabinoside Ara C, daunorubicin DNR, homoharringtonine HHT  [ **ENG** ]   [ **ACVRL1** ]  and hydroxyurea HU, either alone or in combination with imatinib.  Ref: 12454739 Leukemia, 2002
- Hereditary hemorrhagic telangiectasia shows great genetic heterogeneity, and its phenotypes have been classified based on the recently identified mutated genes endoglin  [ **ENG** ]  HHT  [ **ENG** ]   [ **ACVRL1** ]  1 and activin like kinase receptor 1 HHT  [ **ENG** ]  2  [ **ACVRL1** ] .  Ref: 12544200 J Clin Gastroenterol, 2003
- The transforming growth factor beta signaling family is a key player in genetic and multifactorial diseases, including hereditary hemorrhagic telangiectasia HHT  [ **ENG** ]   [ **ACVRL1** ] , cancer, atherosclerosis and immunomodulation.  Ref: 12812985 Hum Mol Genet, 2003
- A Silastic obturator has the advantage of being a non surgical intervention and allows temporary relief of the nasal obstruction.We present the cases of three patientswith HHT  [ **ENG** ]   [ **ACVRL1** ]  who used the obturator with good clinical effect, suggesting that this is a realistic non surgical alternative to Young s procedure for patients with HHT  [ **ENG** ]   [ **ACVRL1** ] .  Ref: 12385360 J Laryngol Otol, 2002
- Boiled plasma prevented the conversion of HHT  [ **ENG** ]   [ **ACVRL1** ]  to HHT  [ **ENG** ]   [ **ACVRL1** ]  acid, suggesting that the conversion was enzymatically mediated.  Ref: 12724858 J Exp Ther Oncol< MedlineTA>J Exp Ther Oncol, 2002
- We present the case of an 82 year old lady with intractable epistaxis secondary to HHT  [ **ENG** ]   [ **ACVRL1** ] , that was successfully controlled by the application of fibrin glue.  Ref: 12437844 J Laryngol Otol, 2002
- We report here a case of HHT  [ **ENG** ]   [ **ACVRL1** ]  associated with cholestasis due to common bile duct stenosis.  Ref: 12783354 Endoscopy, 2003
- To explore the possible actions of endocrine disruptors on the autacoid synthesis in the body, we investigated the effects of nonylphenol NP, bisphenol A BPA, di n butyl phthalate DBP, benzyl n butyl phthalate BBP, and di 2 ethylhexyl phthalate DEHP on the formation of 12 lipoxygenase metabolite, 12 HETE, and cyclooxygenase metabolites, TXB 2 and 12 HHT  [ **ENG** ]   [ **ACVRL1** ] , from exogenous arachidonic acid AA in rabbit platelets.  Ref: 12781627 Toxicol Appl Pharmacol< MedlineTA>Toxicol Appl Pharmacol, 2003
- A preliminary investigation was performed to exclude association between the predisposition and certain candidate genes including KDR kinase insert domain receptor, TEK TEK tyrosine kinase endothelial,  **ACVRL1**  activin receptor like kinase 1  [ **ACVRL1** ] ,  **ENG**  endoglin  [ **ENG** ]  and FLT4 fms like tyrosine kinase 4.  Ref: 12752572 Clin Genet, 2003
- High pressure liquid chromatography and mass spectrometry were used to confirm the identity and quantify HHT  [ **ENG** ]   [ **ACVRL1** ]  and its metabolite, HHT  [ **ENG** ]   [ **ACVRL1** ]  acid.  Ref: 12724858 J Exp Ther Oncol< MedlineTA>J Exp Ther Oncol, 2003
- Conclusions Hepatic vascular lesions in HHT  [ **ENG** ]   [ **ACVRL1** ]  are varied, ranging from telangiectasias to large shunts between three vascular channels.  Ref: 12813620 Cardiovasc Intervent Radiol< MedlineTA>Cardiovasc Intervent Radiol, 2003
- Overcorrecting for any bias towards overestimation that would be introduced in excluding non penetrant family members, cerebral haemorrhages were more than 20 times more common in male HHT  [ **ENG** ]   [ **ACVRL1** ]  subjects under the age of 45 years than in the general population standardised ratio 22.99.  Ref: 12754343 J Neurol Neurosurg Psychiatry, 2003
- The future objectives of the HHT  [ **ENG** ]   [ **ACVRL1** ]  Center of Bari are to increase knowledge of the disease, to cooperate with other centers with the aim of increasing the number of patients studied and to avoid the limits of therapeutic and diagnostic protocols of a rare disease such as HHT  [ **ENG** ]   [ **ACVRL1** ] .  Ref: 12402665 Ann Ital Med Int< MedlineTA>Ann Ital Med Int, 2003
- SETTING HHT  [ **ENG** ]   [ **ACVRL1** ]  clinic at university teaching hospital.  Ref: 12576351 Chest, 2003
- Liver involvement is reported in up to 30% of persons affected by HHT  [ **ENG** ]   [ **ACVRL1** ] .  Ref: 12544200 J Clin Gastroenterol, 2003
- The diagnosis of HHT  [ **ENG** ]   [ **ACVRL1** ]  consists of recurrent epistaxis, mucocutaneous telangiectasis, visceral vascular lesion and familial occurrence.  Ref: 12636208 Zhonghua Yi Xue Za Zhi Taipei , 2002
- Similar to HHT  [ **ENG** ]   [ **ACVRL1** ]  patients, the mice also exhibited gastrointestinal bleeding, as evidenced by positive fecal occult blood tests.  Ref: 12588795 Hum Mol Genet, 2003
- When mice were given an intravenous i.v. injection of HHT  [ **ENG** ]   [ **ACVRL1** ]  4 mg kg, the HHT  [ **ENG** ]   [ **ACVRL1** ]  acid metabolite was found in both plasma and urine.  Ref: 12724858 J Exp Ther Oncol< MedlineTA>J Exp Ther Oncol, 2003
- These findings stress the need for an early diagnosis of HHT  [ **ENG** ]   [ **ACVRL1** ]  that can be useful for the early control of associated visceral involvement.  Ref: 12843319 J Med Genet< MedlineTA>J Med Genet, 2003
- CONCLUSIONS These data contradict accepted wisdom in many countries that asymptomatic HHT  [ **ENG** ]   [ **ACVRL1** ]  patients are at a low and acceptable risk of haemorrhage.  Ref: 12754343 J Neurol Neurosurg Psychiatry, 2003
- Hereditary hemorrhagic telangiectasia HHT  [ **ENG** ]   [ **ACVRL1** ] , also known as Osler Weber Rendu disease, is a rare disorder characterized by arteriovenous communications in visceral organs.  Ref: 12636208 Zhonghua Yi Xue Za Zhi Taipei , 2002
- The TXAS 2C5 chimera contains heme at nearly a 1 1 molar ratio and catalyzes the formation of TXA 2, MDA, and HHT  [ **ENG** ]   [ **ACVRL1** ]  at a 1 1 1 ratio, although with a reduced catalytic activity compared to wild type TXAS.  Ref: 12859980 Arch Biochem Biophys, 2003
- We found that ALK 1  [ **ACVRL1** ]  specifically upregulated Smad6, Smad7, Id1, Id2, endoglin  [ **ENG** ] , STAT1, and interleukin 1 receptor like 1.  Ref: 12384983 J Cell Physiol, 2002
- A threefold and ninefold resistance to HHT  [ **ENG** ]   [ **ACVRL1** ]  and CET, respectively, was found in CEM ADR5000 cells, while the other MDR cell lines did not show cross resistance compared to their drug sensitive counterparts.  Ref: 12616342 Naunyn Schmiedebergs Arch Pharmacol, 2003
- CONCLUSIONS Some HHT  [ **ENG** ]   [ **ACVRL1** ]  patients with GI bleeding improve on drug therapies, but others fail.  Ref: 12526937 Am J Gastroenterol, 2003
- 12 HHT  [ **ENG** ]   [ **ACVRL1** ] , 13 78% inhibition and weaker inhibition on the formation of 12 HETE 0 49% inhibition.  Ref: 12781627 Toxicol Appl Pharmacol< MedlineTA>Toxicol Appl Pharmacol, 2003
- The present investigation presents a starting point to dissect the genes and molecular pathways involved in the tumor cells response to CET and HHT  [ **ENG** ]   [ **ACVRL1** ]  in greater detail.  Ref: 12616342 Naunyn Schmiedebergs Arch Pharmacol, 2003
- K562 cell line was used to observe the effects of HHT  [ **ENG** ]   [ **ACVRL1** ]  on the induction of apoptosis and on the expression of the specific chimeric protein P210 bcr abl, as evaluated by flow cytometric annexin V PI dual labeling technique and Western blot.  Ref: 12578668 Zhongguo Shi Yan Xue Ye Xue Za Zhi< MedlineTA>Zhongguo Shi Yan Xue Ye Xue Za Zhi, 2003
- The results indicated that compared with the control, the level of hTERT mRNA synthesis in HL 60 cells treated with HHT  [ **ENG** ]   [ **ACVRL1** ]  at 0.005 0.03 micro g ml for 12 hours did not change significantly, but it reduced obviously at 0.04 micro g ml and was undetectable at 0.05 micro g ml.  Ref: 12744738 Zhongguo Shi Yan Xue Ye Xue Za Zhi, 2003
- Rendu Osler Weber disease, or hereditary hemorrhagic telangiectasia HHT  [ **ENG** ]   [ **ACVRL1** ] , is an autosomal dominant disease characterized by systemic vascular dysplasia.  Ref: 12402665 Ann Ital Med Int< MedlineTA>Ann Ital Med Int, 2003
- This case demonstrates that HHT  [ **ENG** ]   [ **ACVRL1** ]  may coexist with a primary malignancy and mimic multiple metastases.  Ref: 12517049 J Formos Med Assoc, 2002
- A mouse model for hereditary hemorrhagic telangiectasia HHT  [ **ENG** ]   [ **ACVRL1** ]  type 2.  Ref: 12588795 Hum Mol Genet, 2003
- The aim of this report is to highlight the biliary manifestations of HHT  [ **ENG** ]   [ **ACVRL1** ]  and draw attention to an unusual complication of ERCP in this setting.  Ref: 12783354 Endoscopy, 2003
- Haemorrhages were also six times more common in female HHT  [ **ENG** ]   [ **ACVRL1** ]  subjects 6.18.  Ref: 12754343 J Neurol Neurosurg Psychiatry, 2003
- METHODS Retrospective study on stroke incidence in individuals with HHT  [ **ENG** ]   [ **ACVRL1** ]  and their immediate families n = 674.  Ref: 12754343 J Neurol Neurosurg Psychiatry, 2003
- From September 2000 to March 200 00 patients 63 males, 37 females, mean age 45.5 17.3 years potentially affected by HHT  [ **ENG** ]   [ **ACVRL1** ]  were evaluated in the HHT  [ **ENG** ]   [ **ACVRL1** ]  Center of the Augusto Murri Internal Medicine Section at the University of Bari on a day hospital or hospitalization basis.  Ref: 12402665 Ann Ital Med Int< MedlineTA>Ann Ital Med Int, 2003
- Vascular lesions of the gastrointestinal GI tract include arterio venous malformations as angiodysplasia and Dieulafoy s lesion, venous ectasias multiple phlebectasias and haemorroids, teleangiectasias which can be associated with hereditary hemorrhagic teleangiectasia HHT  [ **ENG** ]   [ **ACVRL1** ] , Turner s syndrome and systemic sclerosis, haemangioma s, angiosarcoma s and disorders of connective tissue affecting blood vessels as pseudoxanthoma elasticum and Ehlers Danlos s disease.  Ref: 12619428 Acta Gastroenterol Belg< MedlineTA>Acta Gastroenterol Belg, 2003
- Because heterozygous mice contain the appropriate genotype for an animal model of this disorder, mice heterozygous for a loss of function mutation in  **Acvrl1**  were carefully examined for an HHT  [ **ENG** ]   [ **ACVRL1** ]  like phenotype.  Ref: 12588795 Hum Mol Genet, 2003
- This controlled, prospective study defined sonographic criteria for hepatic involvement in HHT  [ **ENG** ]   [ **ACVRL1** ] .  Ref: 12717395 Hepatology, 2003
- The diameter of the common hepatic artery was significantly dilated without overlap between HHT  [ **ENG** ]   [ **ACVRL1** ]  patients with liver involvement and the 3 control groups mean 11.3 2.8 mm HHT  [ **ENG** ]   [ **ACVRL1** ]  with liver involvement, 4.6 0.9 mm HHT  [ **ENG** ]   [ **ACVRL1** ]  without liver involvement, 4.8 1.0 mm cirrhosis, and 4.4 1.0 mm healthy controls, P < ;.  Ref: 12717395 Hepatology, 2003
- Hereditary hemorrhagic telangiectasia HHT  [ **ENG** ]   [ **ACVRL1** ] , or Rendu Osler Weber syndrome, is a rare disorder that is closely linked to the development of pulmonary arteriovenous malformations PAVM.  Ref: 12568707 Arch Bronconeumol, 2002
- During a mean follow up of 18.9 months, the group of 40 patients with HHT  [ **ENG** ]   [ **ACVRL1** ]  related bleeding had improvements in their mean Hb and blood transfusion requirements.  Ref: 12526937 Am J Gastroenterol, 2003
- These mice developed age dependent vascular lesions in the skin, extremities, oral cavity and in the internal organs lung, liver, intestine, spleen and brain, similar to those seen in HHT  [ **ENG** ]   [ **ACVRL1** ]  patients.  Ref: 12588795 Hum Mol Genet, 2003
- Hepatobiliary manifestations of hereditary hemorrhagic telangiectasia HHT  [ **ENG** ]   [ **ACVRL1** ]  are rare, but often involve cholestasis.  Ref: 12783354 Endoscopy, 2003
- When HL 60 cells incubated with HHT  [ **ENG** ]   [ **ACVRL1** ]  at 0.02 micro g ml for various hours, the expression of hTERT mRNA did not decrease after 6 18 hours and reduced significantly after 24 hours and was undetectable at 30 hours.  Ref: 12744738 Zhongguo Shi Yan Xue Ye Xue Za Zhi, 2003
- A variety of therapies have been proposed for epistaxis control in HHT  [ **ENG** ]   [ **ACVRL1** ]  but with limited success.  Ref: 12658738 Head Neck, 2003
- BACKGROUND The frequency of haemorrhage in individuals with hereditary haemorrhagic telangiectasia HHT  [ **ENG** ]   [ **ACVRL1** ] , 10% of whom will have cerebral arteriovenous AV malformations, could be high enough to justify screening.  Ref: 12754343 J Neurol Neurosurg Psychiatry, 2003
- 22 061 HHT  [ **ENG** ]   [ **ACVRL1** ]  patient years, specifically analysing patients under 46 years of age 17 515 patient years.  Ref: 12754343 J Neurol Neurosurg Psychiatry, 2003
- Thus, CET and HHT  [ **ENG** ]   [ **ACVRL1** ]  were more active in cell lines without p53 mutation.  Ref: 12616342 Naunyn Schmiedebergs Arch Pharmacol, 2003
- Several agents are being investigated for treatment of advanced phase CML, including decitabine DAC, homoharringtonine HHT  [ **ENG** ]   [ **ACVRL1** ] , troxacitabine, clofarabine, farnesyl transferase FTase inhibitors FTI, and others.  Ref: 12563614 Semin Hematol, 2003
- Non HHT  [ **ENG** ]   [ **ACVRL1** ]  related causes of GI bleeding were diagnosed in four patients.  Ref: 12526937 Am J Gastroenterol, 2003
- Color Doppler sonography and pulsed Doppler sonography were used to study 25 patients with HHT  [ **ENG** ]   [ **ACVRL1** ]  and liver involvement, 20 patients with HHT  [ **ENG** ]   [ **ACVRL1** ]  without liver involvement, 25 patients with cirrhosis, and 25 patients without liver disease.  Ref: 12717395 Hepatology, 2003
- It could be promising that HHT  [ **ENG** ]   [ **ACVRL1** ]  be used extensively in the chemotherapy of patients with CML.  Ref: 12578668 Zhongguo Shi Yan Xue Ye Xue Za Zhi< MedlineTA>Zhongguo Shi Yan Xue Ye Xue Za Zhi, 2003
- Autosomal dominant hereditary haemorrhagic telangiectasia HHT  [ **ENG** ]   [ **ACVRL1** ]  is a genetically heterogeneous disease caused by mutations in at least two different loci.  Ref: 12786761 Clin Genet< MedlineTA>Clin Genet, 2003
- Hereditary haemorrhagic telangiectasia HHT  [ **ENG** ]   [ **ACVRL1** ]  is a genetic vascular disorder characterised by epistaxis, telangiectases, and visceral manifestations.  Ref: 12843319 J Med Genet< MedlineTA>J Med Genet, 2003
- The amount of P210 bcr abl oncoprotein was decreased by approximately 70% when the cells were exposed to HHT  [ **ENG** ]   [ **ACVRL1** ]  for 48 hours that of its partner P145 c abl proto oncoprotein was not affected.  Ref: 12578668 Zhongguo Shi Yan Xue Ye Xue Za Zhi< MedlineTA>Zhongguo Shi Yan Xue Ye Xue Za Zhi, 2003
- The relationship between regulation of hTERT mRNA expression and HHT  [ **ENG** ]   [ **ACVRL1** ]  induced apoptosis is worth investigating furtherly.  Ref: 12744738 Zhongguo Shi Yan Xue Ye Xue Za Zhi, 2003
- CONCLUSION The combined treatment regimen with APC and topically applied estriol provides a new treatment concept of epistaxis in HHT  [ **ENG** ]   [ **ACVRL1** ] .  Ref: 12589417 HNO, 2003
- The prevalence of liver involvement of HHT  [ **ENG** ]   [ **ACVRL1** ]  was reported to range from 8 to 31%.  Ref: 12636208 Zhonghua Yi Xue Za Zhi Taipei , 2002
- HHT  [ **ENG** ]   [ **ACVRL1** ]  was diagnosed in 112 individuals 51%, with at least a point prevalence of 1 in 31 inhabitants of Curaçao and Bonaire older than twelve years.  Ref: 12522784 Am J Med Genet, 2003
- Hereditary hemorrhagic telangiectasia HHT  [ **ENG** ]   [ **ACVRL1** ]  is an autosomal dominant disorder characterized by the age dependent development of focal arteriovenous malformations and telangiectases.  Ref: 12588795 Hum Mol Genet, 2003
- In all patients with HHT  [ **ENG** ]   [ **ACVRL1** ]  and liver involvement, areas with intrahepatic hypervascularization caused by dilated intrahepatic arteries were observed in varying intensity.  Ref: 12717395 Hepatology, 2003
- The tendency for suppression of telomerase activity was consistent with decrease of the expression of hTERT mRNA in HHT  [ **ENG** ]   [ **ACVRL1** ]  treated HL 60 cells.  Ref: 12744738 Zhongguo Shi Yan Xue Ye Xue Za Zhi, 2003
- The initial half lives t 1 2 alpha of HHT  [ **ENG** ]   [ **ACVRL1** ]  and HHT  [ **ENG** ]   [ **ACVRL1** ]  acid were 9 and 17 min, respectively.  Ref: 12724858 J Exp Ther Oncol< MedlineTA>J Exp Ther Oncol, 2003
- In conclusion, the diameter of the common hepatic artery >7 mm and intrahepatic hypervascularization are suitable sonographic diagnostic parameters of HHT  [ **ENG** ]   [ **ACVRL1** ]  with high sensitivity and specificity.  Ref: 12717395 Hepatology, 2003
- Hereditary haemorrhagic telangiectasia HHT  [ **ENG** ]   [ **ACVRL1** ]  is an autosomal dominant disorder characterized by dermal, mucosal, and visceral telangiectases as well as pulmonary and cerebral arteriovenous malformations.  Ref: 12437844 J Laryngol Otol, 2002
- By clinical history and physical examination, 219 persons over age twelve with a first degree relative with HHT  [ **ENG** ]   [ **ACVRL1** ]  were screened.  Ref: 12522784 Am J Med Genet, 2003
- The lethal dose of HHT  [ **ENG** ]   [ **ACVRL1** ]  affecting 50% LD50 of mice was 6.7 mg kg, but HHT  [ **ENG** ]   [ **ACVRL1** ]  acid produced no apparent toxic effects at doses up to 280 mg kg.  Ref: 12724858 J Exp Ther Oncol< MedlineTA>J Exp Ther Oncol, 2003
- Although ALK 1  [ **ACVRL1** ]  and another endothelial specific TGF beta receptor, endoglin  [ **ENG** ] , both bind TGF beta with identical isoform specificity and form a complex together, neither has been shown to signal in response to TGF beta, and the mechanism by which these receptors signal in endothelial cells remains unknown.  Ref: 12393874 J Biol Chem, 2002
- Hereditary hemorrhagic telangiectasia HHT  [ **ENG** ]   [ **ACVRL1** ] , also known as Osler Weber Rendu disease, is an hereditary disorder that results in fibrovascular dysplasia with the development of telangiectasias and arteriovenous malformations.  Ref: 12544200 J Clin Gastroenterol, 2003
- Epistaxis associated with hereditary haemorrhagic telangiectasia HHT  [ **ENG** ]   [ **ACVRL1** ]  is a challenging condition.  Ref: 12385360 J Laryngol Otol, 2002
- Other families with phenotypic HHT  [ **ENG** ]   [ **ACVRL1** ]  do not bear these mutations.  Ref: 12544200 J Clin Gastroenterol, 2003
- Herein, we present a 75 year old male who was diagnosed as having HHT  [ **ENG** ]   [ **ACVRL1** ]  with liver involvement, based on the findings of recurrent epistaxis, mucosal telangiectasis on the lower lip and hepatic arteriovenous malformation.  Ref: 12636208 Zhonghua Yi Xue Za Zhi Taipei , 2002
- Methods We reviewed the angiographic findings of seven patients with HHT  [ **ENG** ]   [ **ACVRL1** ] .  Ref: 12616420 Cardiovasc Intervent Radiol< MedlineTA>Cardiovasc Intervent Radiol, 2002
- This review focuses on the evolving evidence base for HHT  [ **ENG** ]   [ **ACVRL1** ]  management, issues regarding pregnancy and prothrombotic treatments, and discusses the molecular and cellular changes that underlie this disease.  Ref: 12566546 Postgrad Med J, 2003
- To investigate the mechanism and its significance of the depression of telomerase activity in HL 60 cells exposed to homoharringtonine HHT  [ **ENG** ]   [ **ACVRL1** ] , the semi quntitative RT PCR and PCR ELISA were used to detect the expression of hTERT mRNA and telomerase activity in HL 60 cells, the apoptotic rate of HL 60 cells was assayed with TUNEL.  Ref: 12744738 Zhongguo Shi Yan Xue Ye Xue Za Zhi, 2003
- The diagnosis of HHT  [ **ENG** ]   [ **ACVRL1** ]  was confirmed in 56 patients and suspected in 10.  Ref: 12402665 Ann Ital Med Int< MedlineTA>Ann Ital Med Int, 2003
- For the first time the parental germline in which a de novo HHT  [ **ENG** ]   [ **ACVRL1** ]  mutation occurred has been identified.  Ref: 12786761 Clin Genet< MedlineTA>Clin Genet, 2003
- U 87MG.DeltaEGFR cells transfected with an epidermal growth factor receptor gene truncated in its extracellular domain through a deletion of exons 2 7 were 14 fold more resistant to HHT  [ **ENG** ]   [ **ACVRL1** ]  than control cells transfected with mock expression vector or non transfected cells.  Ref: 12616342 Naunyn Schmiedebergs Arch Pharmacol, 2003
- The alpha L arabinofuranosidase AF from the fungus Rhizomucor pusillus HHT  [ **ENG** ]   [ **ACVRL1** ]  1 released arabinose at appreciable rates from 1 >5 alpha L arabinofuranooligosaccharides, sugar beet arabinan and debranched arabinan.  Ref: 12829392 Carbohydr Res, 2003
- Sonographic criteria for the diagnosis of hepatic involvement in hereditary hemorrhagic telangiectasia HHT  [ **ENG** ]   [ **ACVRL1** ] .  Ref: 12717395 Hepatology, 2003
- As the tumor suppressor p53 is another important factor of chemoresistance, we also analyzed the possibility that p53 affects the response of tumor cells to CET and HHT  [ **ENG** ]   [ **ACVRL1** ] .  Ref: 12616342 Naunyn Schmiedebergs Arch Pharmacol, 2003
- It is clear from the study that HHT  [ **ENG** ]   [ **ACVRL1** ]  is an inhibitor of P210 bcr abl oncoprotein and therefore promotes the apoptosis of CML cells.  Ref: 12578668 Zhongguo Shi Yan Xue Ye Xue Za Zhi< MedlineTA>Zhongguo Shi Yan Xue Ye Xue Za Zhi, 2003
- OBJECTIVES To define the annual incidence of haemorrhagic stroke in individuals with HHT  [ **ENG** ]   [ **ACVRL1** ] .  Ref: 12754343 J Neurol Neurosurg Psychiatry, 2003
- HHT  [ **ENG** ]   [ **ACVRL1** ]  can be definitely diagnosed with the presence of all these three criteria.  Ref: 12636208 Zhonghua Yi Xue Za Zhi Taipei , 2002
